# Laser Remelting of Biocompatible Ti-Based Glass-Forming Alloys: Microstructure, Mechanical Properties, and Cytotoxicity

**DOI:** 10.3390/ma18245687

**Published:** 2025-12-18

**Authors:** Aleksandra Małachowska, Wiktoria Drej, Agnieszka Rusak, Tomasz Kozieł, Denis Pikulski, Wojciech Stopyra

**Affiliations:** 1 Faculty of Mechanical Engineering, Wroclaw University of Science and Technology, ul. Lukasiewicza 5, 50-371 Wroclaw, Polandwojciech.stopyra@pwr.edu.pl (W.S.); 2 Center for Materials Engineering and Metal Forming, Wroclaw University of Science and Technology, Wybrzez Wyspianskiego 27, 50-370 Wroclaw, Poland; 3 Division of Histology and Embryology, Department of Human Morphology and Embryology, Wroclaw Medical University, Tytusa Chalubinskiego 6a, 50-368 Wroclaw, Poland; agnieszka.rusak@umw.edu.pl; 4 Faculty of Metals Engineering and Industrial Computer Science, AGH University of Krakow, Al. Mickiewicza 30, 30-059 Krakow, Poland; tkoziel@agh.edu.pl (T.K.); dapikulski@agh.edu.pl (D.P.)

**Keywords:** bulk metallic glasses (BMGs), titanium alloys, biocompatibility

## Abstract

Titanium-based bulk metallic glasses (BMGs) offer high strength, lower stiffness than Ti-6Al-4V, and superior corrosion resistance, but conventional Ti glass-forming systems often contain toxic Ni, Be, or Cu. This work investigates five novel Ti-based alloys free of these elements—Ti_42_Zr_35_Si_5_Co_12.5_Sn_2.5_Ta_3_, Ti_42_Zr_40_Ta_3_Si_15_, Ti_60_Nb_15_Zr_10_Si_15_, Ti_39_Zr_32_Si_29_, and Ti_65.5_Fe_22.5_Si_12_—synthesized by arc melting and suction casting. Single-track laser remelting using a selective laser melting (SLM) system was performed to simulate additive manufacturing and examine microstructural evolution, cracking behavior, mechanical properties, and cytocompatibility. All alloys solidified into fully crystalline α/β-Ti matrices with Ti/Zr silicides; no amorphous structures were obtained. Laser remelting refined the microstructure but did not induce glass formation, consistent with the known limited glass-forming ability of Cu/Ni/Be-free Ti systems. Cracking was observed at low laser energies but crack density decreased as laser energy increased. Cracks were eliminated above ~0.4 J/mm for most alloys. Ti_42_Zr_35_Si_5_Co_12.5_Sn_2.5_Ta_3_ exhibited the lowest stiffness (~125 GPa), while Ti_60_Nb_15_Zr_10_Si_15_ showed the highest due to silicide precipitation. Cytotoxicity tests (ISO 10993-5) confirmed all alloys to be non-toxic, with some extracts even enhancing fibroblast proliferation. This rapid laser-remelting approach enables cost-effective screening of Ti-based glass-forming alloys for additive manufacturing. Ti–Zr–Ta–Si systems demonstrated the most promising properties for further testing using the powder bed method.

## 1. Introduction

Titanium alloys have long been used as materials for orthopedic implants, especially due to their favorable mechanical strength and biocompatibility. However, the most popular alloy, Ti-6Al-4V, which was initially developed for mechanical applications in aerospace engineering [1], presents several drawbacks. The elastic modulus of human bone depends on its mineral density and microstructure [2]. For example, cancellous bone exhibits an elastic modulus in the range of 0.1–1.1 GPa [3] or 0.2–4 GPa [2]. For cortical bone, reported values are approximately 20.15 ± 3.51 GPa for interstitial tissue and 16.50 ± 3.46 GPa [4] and ~14–15 GPa [5] for osteonal tissue. These values are much lower than those for the commonly used Ti-6Al-4V, which exhibits an elastic modulus of 111–117 GPa for both the additively manufactured and conventionally hot-formed versions [6]. This considerable disparity leads to a phenomenon known as stress shielding. In this scenario, the stiffer implant bears a disproportionate amount of the physiological load, resulting in insufficient mechanical stimulation and load transfer to the adjacent bone tissue. Consequently, bone resorption may occur, ultimately leading to implant loosening and failure and the need for costly and invasive revision surgeries [7]. Attempts to mitigate this problem have focused on introducing controlled porosity to improve load transfer [8]. Another critical issue for Ti-6Al-4V alloys is the release of potentially toxic metallic ions and/or particles into the physiological environment. Implant degradation occurs through various mechanisms, including stress corrosion, pitting corrosion, and wear [9], generating micro- and nanoparticles as well as metallic ions [10]. For example, vanadium ions have been reported to be released from Ti-6Al-4V alloys [11]. Chronic aluminum (Al) accumulation may contribute to neurodegeneration, renal dysfunction, anemia, and osteomalacia, while excessive vanadium (V) levels have been linked to systemic toxicity, including neuropathy and renal and reproductive damage. Therefore, these elements should be eliminated from implant materials [12].

Osteolysis and aseptic loosening account for approximately 75% of implant failures, with metal ions contributing to this process by elevating key inflammatory cytokine levels that subsequently activate osteoclasts [13]. Therefore, new titanium alloy compositions are being developed to address these concerns [14]. One promising direction involves titanium-based bulk metallic glasses (BMGs), which have been investigated as potential implant materials due to their superior mechanical properties, including high strength, lower Young’s modulus (~90 GPa) compared to Ti-6Al-4V (~115 GPa) [15], and enhanced corrosion resistance [16].

The main drawback is that most glass-forming Ti alloy compositions include cytotoxic elements such as Ni, Be, or Cu, which improve the glass-forming ability (GFA) [17]. The highest critical diameters are reported for Ti alloys containing Be, reaching even above 50 mm [18]. Other systems, such as Ti–Zr–Cu–Pd or Ti–Zr–Cu–Pd–Sn, can form glass with diameters of up to 6 mm and 10 mm, respectively [19,20]. However, the use of Pd significantly increases the alloy cost due to its noble character. Elements that are both biocompatible and favorable for glass formation in Ti systems are B, Si, P, and In [17]. In addition to incorporating biocompatible glass formers, promising Ti-based BMGs should contain highly biocompatible β-isomorphous elements such as Nb, Zr, and/or Ta, which also exhibit low metal ion release [17]. Taking these criteria into account, the range of potential biocompatible alloy compositions is limited. Therefore, some proposed alloys still contain such elements as iron [21] or copper [22].

Most of the reported Ti-based BMG compositions have been produced only in limited geometries, such as ribbons [16,23], thin films [24], or small spark plasma sintered (SPS) samples [25]. One of the modern approaches to the production of customized implants is additive manufacturing (AM). The process parameters and properties of AM-fabricated Ti-6Al-4V components have been extensively studied [26,27,28]. However, there are relatively few studies concerning the 3D printing of Ti-based metallic glass alloys [29,30].

Deng et al. [29] successfully fabricated fully glassy samples of the TiCu_38_Zr_7.5_Fe_2.5_Sn_2_Si_1_Ag_2_ alloy using selective laser melting (SLM), demonstrating for the first time the feasibility of producing Ti-based bulk metallic glasses (BMGs) via this additive manufacturing method. The SLM process eliminated crystalline phases present in the precursor powder, resulting in homogeneous amorphous structures with high relative densities exceeding 98.5%. Despite exhibiting slightly lower compressive strength (~1700 MPa) compared to as-cast rods (~2000 MPa), the SLM samples retained high thermal stability and characteristic glass transition behavior, indicating that the elevated oxygen content introduced during processing did not impair their glass-forming ability [29]. However, the high Cu content in that alloy raises questions about cytotoxicity.

Chen et al. [30] developed a novel biocompatible Ti-based bulk metallic glass composite (BMGC) with high glass-forming ability (GFA), specifically Ti_42_Zr_35_Si_5_Co_12.5_Sn_2.5_Ta_3_, and successfully fabricated it using selective laser melting (SLM). Despite optimizing the alloy composition based on eutectic and atomic size criteria to achieve superior thermal stability without Cu, the produced powder was only partially amorphous. The obtained SLM prints were dense, containing both amorphous and crystalline phases [30]. In this article, five biocompatible Ti-based metallic glass compositions without Cu were examined. The objective of this study was to evaluate the processability of Ti-based alloys using single-track laser remelting, with focus on microstructural evolution, cracking susceptibility, and evaluating whether the applied remelting conditions could induce amorphization. Although this method does not replicate full layer-by-layer interactions typical of additive manufacturing, it provides a rapid and cost-effective approach for identifying compositions suitable for further development. The findings show that, despite the use of biocompatible elements, the investigated Ti-based alloys did not form amorphous structures under the applied conditions, which limits their suitability for producing bulk metallic glasses using additive techniques such as selective laser melting (SLM).

## 2. Materials and Methods

Based on the literature review, five biocompatible Ti-based alloys free of Be, Cu, and rare-earth elements were selected for experimental validation: Ti_42_Zr_35_Si_5_Co_12.5_Sn_2.5_Ta_3_ [30], Ti_42_Zr_40_Ta_3_Si_15_ [31,32], Ti_60_Nb_15_Zr_10_Si_15_ [33], Ti_39_Zr_32_Si_29_, and Ti_65.5_Fe_22.5_Si_12_ [21]. Alloys were synthesized by arc melting followed by suction casting to produce cylindrical rods. High-purity elemental Ti, Zr, Co, Nb, Fe, Ta, Sn, and Si (≥99.9%) (Onyxmet Tomasz Olszewski, Olsztyn, Poland) were accurately weighed and melted using an Arc Melter (Edmund Bühler GmbH, Bodelshausen, Germany) under a purified argon atmosphere with a titanium getter. To ensure chemical homogeneity, each ingot was remelted four times. Subsequently, the molten alloys were suction-cast into a water-cooled copper mold with a 3 mm diameter cavity to form rods. The Ti_42_Zr_35_Si_5_Co_12.5_Sn_2.5_Ta_3_ alloy exhibited low melt fluidity and did not completely fill the 3 mm mold; therefore, a mold with a larger cavity (5 mm in diameter) was used for this composition. After casting, the rods were sectioned into 4 mm long cylinders using a wire EDM machine. The samples were then mounted in aluminum disks with drilled holes to provide a flat surface for laser remelting using a selective laser melting (SLM) system. The exposed surfaces were ground using SiC papers up to #1200 grit to obtain a smooth, flat surface and to ensure an identical focal plane for all specimens. The number of usable samples varied depending on the yield of sound rods obtained after casting. In some cases, the suction-cast rods were partially hollow, forming thin-walled tubes that could not be used for subsequent laser remelting.

### 2.1. SLM Printing

To preliminarily assess the printability of the selected titanium-based alloy compositions, surface laser-remelting experiments were performed using a Nikon SLM Solutions 280 2.0 (SLM Solutions Group AG, Lübeck, Germany) system to analyze cracking susceptibility and thermal response. This step simulated the conditions of laser-based additive manufacturing. During the remelting process, the chamber was purged with high-purity argon to minimize oxidation. The parameters varied were laser power (300–400 W) and scanning speed (600–1400 mm/s), while the laser spot size (100 µm) and hatch spacing (0.12 mm) were kept constant. The linear energy density (*ED*) was calculated using Equation (1):

(1) $$ED=\frac{P}{v}$$

where *P* is laser power [W] and *v* is scanning speed [mm/s]. Key processing parameters and *ED* values are summarized in Table 1.

### 2.2. Microstructural Characterization

Phase analysis of the as-cast samples was performed using an X-ray diffractometer (Bruker AXS D8 Advance, Bruker AXS, Karlsruhe, Germany; Cu Kα radiation) to identify phase constituents and detect possible amorphous phase formation (broad halo). The diffraction patterns were collected over a 2θ range of 20–70° with a step size of 0.02°. The microstructure was examined using a scanning electron microscope (SEM, Tescan Vega 3, Tescan Orsay Holding, Brno-Kohoutovice, Czech Republic) operated at an accelerating voltage of 10 kV. The crack density in the remelted regions was assessed from SEM images using ImageJ software (1.54k). The crack density is calculated as the total crack line length divided by the area of the remelted region (units of mm^−1^) according to Equation (2):

(2) $$L_{crack}=\frac{\mathrm{Total}\;length\;of\;cracks\;[mm]}{Area\;of\;remelted\;\mathrm{surface}\;[{mm}^{2}]}$$

### 2.3. Mechanical Testing (Nanoindentation)

The hardness and elastic modulus were measured by instrumented nanoindentation on the sample surfaces. A Berkovich diamond indenter was used with a maximum load of 10 mN and a loading/unloading rate of 20 mN/min. Indents were placed at a minimum distance of three indentation diameters apart to avoid interaction of stress fields. Hardness and indentation modulus were evaluated from the load-displacement curves using the Oliver–Pharr method. A Poisson’s ratio of 0.35 was assumed for the modulus calculations, based on values in the literature for similar Ti-based BMGs. For each condition (as-cast and each laser energy density, *ED*), at least 10 indents were performed and the results averaged. The mechanical properties of the as-cast rods served as a baseline against which the effects of laser remelting were compared.

### 2.4. Cytotoxicity Testing

Cytotoxicity was evaluated in accordance with PN-EN ISO 10993-5:2009 [34]. using two reference murine fibroblast cell lines: L929 and Balb/3T3 (ATCC). The cells were cultured under standard conditions (37 °C, 5% CO_2_, humidified atmosphere) in MEM or DMEM media supplemented with 10% fetal bovine serum (FBS) and antibiotics. Sterile metallic disks (Ø ≈ 5 mm) were used as test specimens.

#### 2.4.1. Indirect Assay

Extracts were prepared at a ratio of 2 g of material per 10 mL of culture medium and tested at 100%, 50%, 25%, and 12.5% concentrations. The negative control consisted of high-density polyethylene (HDPE; United States Pharmacopeia (USP) Reference Standard, Sigma-Aldrich, St. Louis, MO, USA), while the positive control was a sodium lauryl sulfate (SLS; Sigma-Aldrich) solution at concentrations of 0.2, 0.15, 0.1, and 0.05 mg/mL. An additional negative control was a cell culture without contact with any material.

Sample extraction was carried out in a Steri-Cycle 381 incubator (Thermo Scientific, Waltham, MA, USA) at 37 °C for 24 h, following the guidelines of PN-EN ISO 10993-12:2009 [35]. Extracts were prepared from both the materials polymerized 24 h earlier and the materials polymerizing during extraction.

L929 fibroblast cells, after trypsinization with 0.25% Trypsin-EDTA (Sigma-Aldrich), were seeded into 96-well plates (TPP, Trasadingen, Switzerland) at a density of 1 × 10^4^ cells/well. After 24 h of incubation, the medium was removed and replaced with extracts of the tested samples (100%, 50%, 25%, 12.5%) or the respective controls (complete medium, SLS, HDPE).

Following 24 h exposure, fibroblast morphology was assessed using a phase-contrast microscope (CKX53, Olympus, Tokyo, Japan), and cell viability was determined via the MTT assay. Cell viability (V%) was calculated according to Equation (3):

(3) $$V\left[\%\right]=\frac{A_{b}-A_{m}}{A_{s}-A_{m}}\times 100\%$$

where *V*—cell viability (in %); *A_b_*—average absorbance of the tested sample; *A_s_*—average absorbance of the blank sample; *A_m_*—absorbance of the medium.

The results were expressed as a percentage relative to the blank sample (culture treated only with complete medium). The color intensity of the MTT solution, measured spectrophotometrically, was proportional to the number of viable cells and served as an indicator of cytotoxicity in the tested samples.

#### 2.4.2. Direct Contact Assay

Balb/3T3 fibroblast cells (1.5 × 10^5^ cells/well) were cultured to form a confluent monolayer and then exposed to the test samples placed directly on the cell layer for 24 h. Cytotoxicity was evaluated based on cell morphology beneath and surrounding the material. The control group consisted of cells cultured without direct contact with any material.

The degree of cytotoxic response was classified according to PN-EN ISO 10993-5:2009 [34] criteria. Data normality was verified using the Shapiro–Wilk test, and one-way ANOVA followed by Tukey’s post hoc test was applied for statistical comparisons (Statistica 13.3).

## 3. Results and Discussion

### 3.1. Microstructure

All five alloys were successfully cast into rod form with 3 mm diameter, except for Ti_42_Zr_35_Si_5_Co_12.5_Sn_2.5_Ta_3_, which required a larger mold (5 mm) due to its higher melt viscosity. It must be emphasized that none of the investigated alloys exhibited any amorphous halo in the XRD patterns; the material was fully crystalline under all processing conditions. Therefore, the alloys did not show any tendency toward amorphization at the cooling rates achievable in single-pass laser remelting of bulk samples. This is consistent with reports in the litereature, as these compositions were previously studied mainly in ribbon form (Ti_60_Nb_15_Zr_10_Si_15,_ Ti_42_Zr_40_Ta_3_Si_15_), mechanically alloyed powders (Ti_65.5_Fe_22.5_Si_12_), or small samples sintered from such alloys (Ti_39_Zr_32_Si_29_). Evidently, the absence of biotoxic elements such as Cu, Ni, and Be reduces the glass-forming ability of these alloys [17].

(a) Ti_42_Zr_35_Si_5_Co_12.5_Sn_2.5_Ta_3_

**Figure 1:**
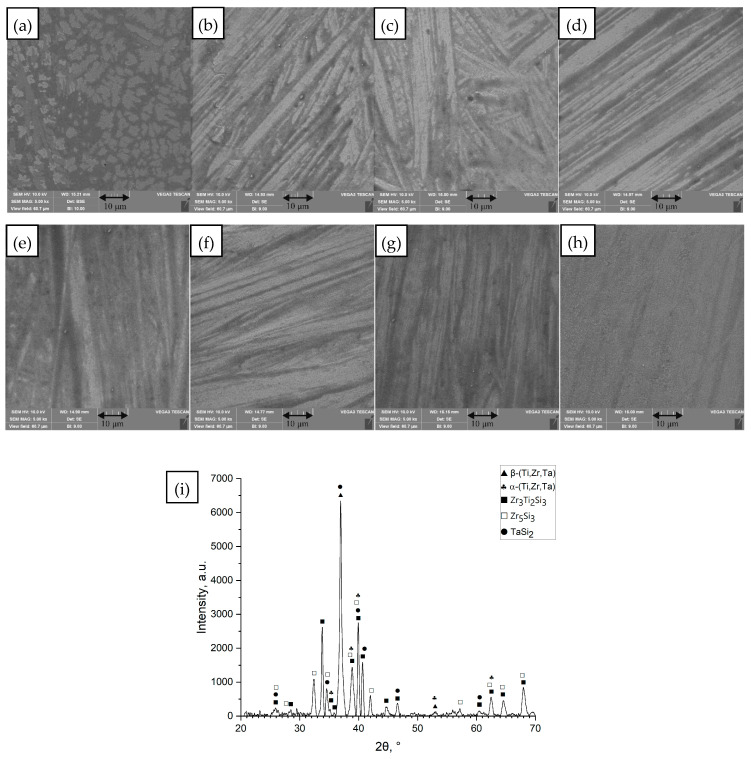
Images of the surface of the unmelted Ti_42_Zr_35_Si_5_Co_12.5_Sn_2.5_Ta_3_ sample and samples remelted by the SLM method at different values of linear energy density (*ED*). (**a**) Unmelted sample; (**b**) *ED* = 0.21 J/mm; (**c**) *ED* = 0.25 J/mm; (**d**) *ED* = 0.29 J/mm; (**e**) *ED* = 0.30 J/mm; (**f**) *ED* = 0.35 J/mm; (**g**) *ED* = 0.40 J/mm; (**h**) *ED* = 0.58 J/mm, processing conditions Table 1 rows 1–8, SEM images (magnification 5000×), (**i**) diffractogram for unmelted sample (**a**).

Figure 1 shows a representative case for the Ti_42_Zr_35_Si_5_Co_12.5_Sn_2.5_Ta_3_ alloy, which was developed by Chen [30] for SLM printing. The SLM printed samples exhibited amorphous matrix with hcp-α-Ti-type nanocrystals. The optimum energy used for SLM printing was 0.2 J/mm with a small overlap of 10%. It was stated that the repeating thermal cycling during laser remelting of each new layer and HAZ zone induce crystallization [30]. In the as-cast condition (Figure 1a), the alloy exhibited a heterogeneous microstructure comprising primarily α′/β Ti-based phases with intermetallic silicide precipitates, including phases such as TaSi_2_, Zr_5_Si_3_, and a mixed (Zr,Ti) silicide (likely Zr_3_Ti_2_Si_3_). After a single laser scan (remelting) on the surface, the microstructure resolidified with a much finer scale. Figure 1b–h correspond to increasing laser energy densities (*ED* = 0.21 up to 0.58 J/mm (Table 1 rows 2–8)). At the lowest ED (0.21 J/mm—Table 1 row 2, Figure 1b), the melt pool was shallow and rapidly solidified. As the energy input increased (Figure 1c–h), the remelted zone became deeper and the thermal gradient shallower, leading to a more columnar, directional microstructure epitaxially growing from the base metal. By *ED* = 0.40 J/mm (Table 1 row 7, Figure 1g) and 0.58 J/mm (Table 1 row 8, Figure 1h), the remelted layer showed a uniform fine architecture. There was no indication that laser remelting had produced any new amorphous phase in Ti_42_Zr_35_Si_5_Co_12.5_Sn_2.5_Ta_3_; rather, it refined the existing crystalline phases.

(b) Ti_42_Zr_40_Ta_3_Si_15_

**Figure 2:**
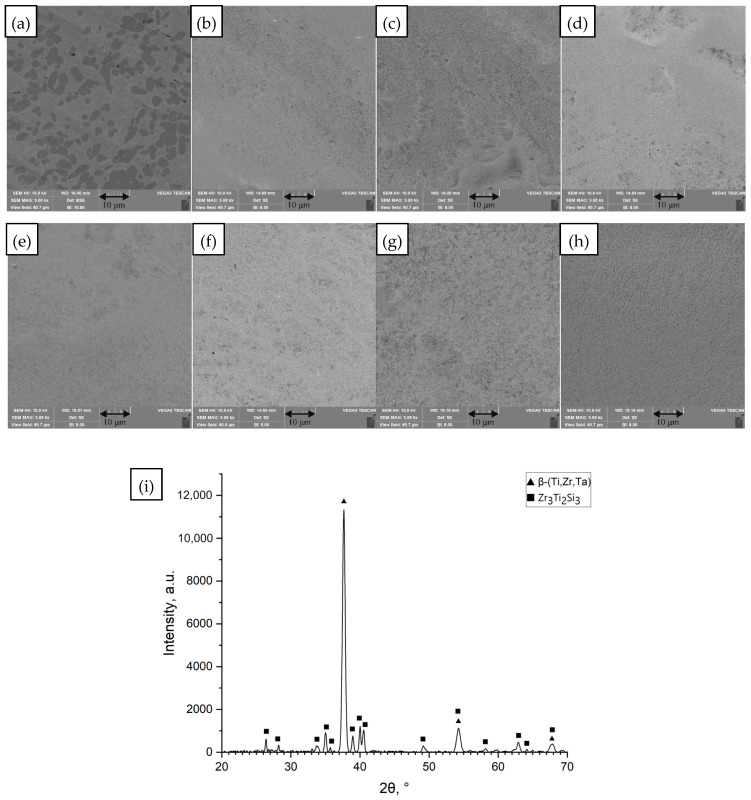
SEM images of the surface of the unmelted Ti_42_Zr_40_Ta_3_Si_15_ sample and samples remelted by the SLM method at different values of linear energy density (*ED*). (**a**) Unmelted sample; (**b**) *ED* = 0.21 J/mm; (**c**) *ED* = 0.25 J/mm; (**d**) *ED* = 0.29 J/mm; (**e**) *ED* = 0.30 J/mm; (**f**) *ED* = 0.35 J/mm; (**g**) *ED* = 0.40 J/mm; (**h**) *ED* = 0.58 J/mm, processing conditions Table 1 rows 1–8, SEM images (magnification 5000×), (**i**) diffractogram for unmelted sample (**a**).

Ti_42_Zr_40_Ta_3_Si_15_ showed a somewhat different microstructure (Figure 2). The Ta addition in this alloy stabilizes the β phase. It is known for its excellent ductility in the amorphous state (capable of 180° bending) [32]. In the as-cast state, there was a more ductile matrix and visible globular precipitates distributed in the matrix. These were likely silicide precipitates in a β-(Ti,Zr,Ta) solid solution matrix. After laser remelting, the precipitates in Ti_42_Zr_40_Ta_3_Si_15_ became significantly finer and more evenly distributed. The overall microstructure after high-*ED* remelting was more homogeneous than the as-cast state. As in Ti_42_Zr_35_Si_5_Co_12.5_Sn_2.5_Ta_3_, no amorphous phase was detected.

(c) Ti_60_Nb_15_Zr_10_Si_15_

**Figure 3:**
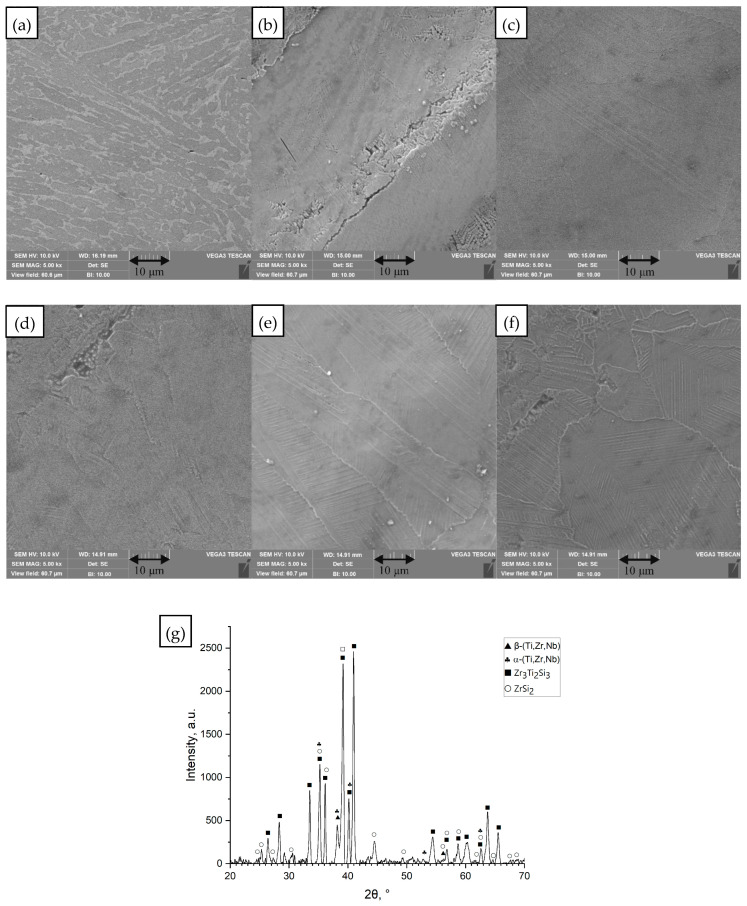
SEM images of the surface of the unmelted Ti_60_Nb_15_Zr_10_Si_15_ sample and samples remelted by the SLM method at different values of linear energy density (*ED*). (**a**) Unmelted sample; (**b**) *ED* = 0.21 J/mm; (**c**) *ED* = 0.25 J/mm; (**d**) *ED* = 0.35 J/mm; (**e**) *ED* = 0.40 J/mm; (**f**) *ED* = 0.58 J/mm, processing conditions Table 1 rows 1–3 and 6–8, SEM images (magnification 5000×), (**g**) diffractogram for unmelted sample (**a**).

The Ti_60_Nb_15_Zr_10_Si_15_ alloy belongs to the Ti–Nb–Zr–Si family of glass-forming systems composed exclusively of biocompatible elements. It should be noted that during suction casting, the Ti_60_Nb_15_Zr_10_Si_15_ alloy exhibited poor mold filling behavior and solidified predominantly as a hollow tube. As a result, the number of usable cylindrical specimens was limited, which restricted the number of laser-remelting conditions that could be tested for this composition. Therefore, not all energy density variants, specifically 0.29 and 0.30 J/mm, were included in the microstructural analysis. The amorphous matrix was stabilized by Nb addition, which increased the glass-forming ability (GFA) by reducing diffusion rates and suppressing heterogeneous nucleation [33]. Prior results on related compositions demonstrated strong cooling-rate dependence of microstructure [33]. In this study, the presence of Nb (a β stabilizer and slow diffuser) did not yield any detectable glass formation under these conditions. In the as-cast condition, the Ti_60_Nb_15_Zr_10_Si_15_ microstructure consisted of a mixture of α- Ti(Nb) and β-Ti(Nb) phases, along with significant volume fractions of silicides (Figure 3). The silicides were identified as Zr_3_Ti_2_Si_3_ and ZrSi_2_. After laser remelting, Ti_60_Nb_15_Zr_10_Si_15_ showed a refined microstructure with silicide precipitates within the Ti,Zr matrix. At intermediate energy densities (0.25–0.35 J/mm, Table 1 rows 3 and 6), a very fine network of silicide needles formed upon rapid solidification. Higher-*ED* remelting (0.58 J/mm, Table 1 row 8) allowed slightly coarser features (because of slower cooling).

(d) Ti_39_Zr_32_Si_29_

**Figure 4:**
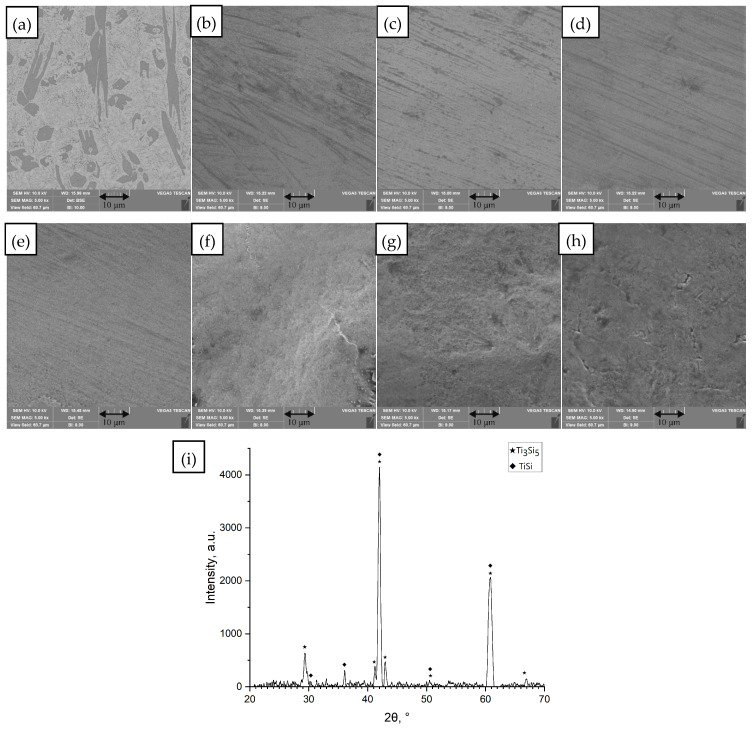
SEM images of the surface of the unmelted Ti_39_Zr_32_Si_29_ sample and samples remelted by the SLM method at different values of linear energy density (ED). (**a**) Unmelted sample; (**b**) *ED* = 0.21 J/mm; (**c**) *ED* = 0.25 J/mm; (**d**) *ED* = 0.29 J/mm; (**e**) *ED* = 0.30 J/mm; (**f**) *ED* = 0.35 J/mm; (**g**) *ED* = 0.40 J/mm; (**h**) *ED* = 0.58 J/mm, processing conditions Table 1 rows 1–8 SEM images (magnification 5000×), (**i**) diffractogram for unmelted sample (**a**).

Ti_39_Zr_32_Si_29_ is a ternary alloy with very high Si content (29 at%). In the as-cast state, Ti_39_Zr_32_Si_29_ was essentially an intermetallic composite consisting predominantly of silicide phases. The microstructure appeared fully crystalline, with a relatively coarse size scale (tens of microns) of silicide domains. After laser remelting (Figure 4), the Ti_39_Zr_32_Si_29_ microstructure became significantly finer.

(e) Ti_65.5_Fe_22.5_Si_12_

**Figure 5:**
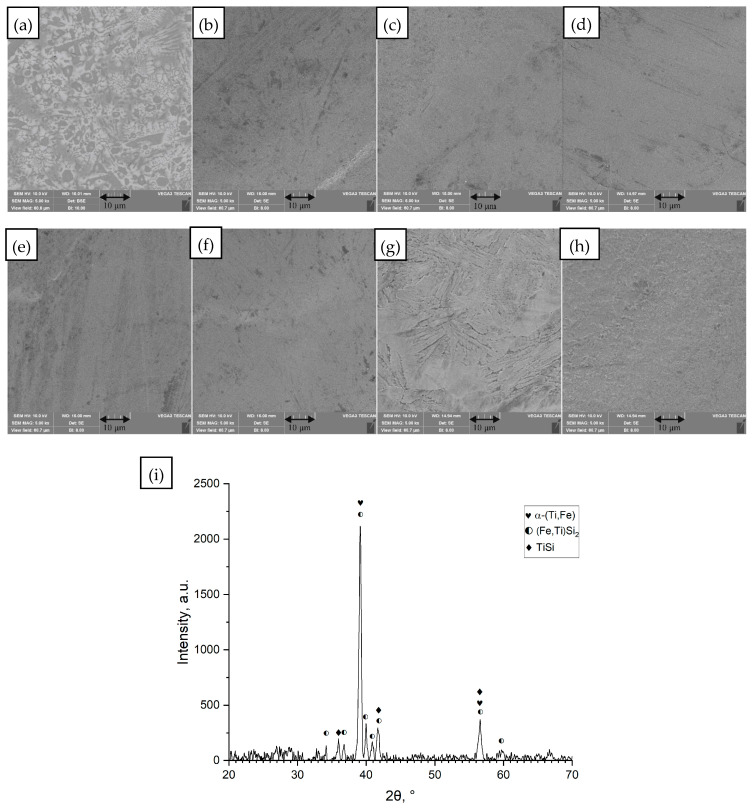
SEM images of the surface of the unmelted Ti_65.5_Fe_22.5_Si_12_ sample and samples remelted by the SLM method at different values of linear energy density (*ED*). (**a**) Unmelted sample; (**b**) *ED* = 0.21 J/mm; (**c**) *ED* = 0.25 J/mm; (**d**) *ED* = 0.29 J/mm; (**e**) *ED* = 0.30 J/mm; (**f**) *ED* = 0.35 J/mm; (**g**) *ED* = 0.40 J/mm; (**h**) *ED* = 0.58 J/mm, processing conditions Table 1 rows 1–8, SEM images (magnification 5000×), (**i**) diffractogram for unmelted sample (**a**).

Ti_65.5_Fe_22.5_Si_12_ belongs to the Ti–Fe–Si family. The as-cast microstructure of Ti_65.5_Fe_22.5_Si_12_ consisted mostly of β-Ti and silicides. After laser remelting (Figure 5), the Ti_65.5_Fe_22.5_Si_12_ microstructure became refined. This refinement is beneficial as it can improve mechanical performance (e.g., toughness) relative to the coarse as-cast state. As with other alloys, no amorphous regions were produced; Ti_65.5_Fe_22.5_Si_12_ remained fully crystalline even at the lowest energies.

To sum up, despite being designed for high glass-forming ability (GFA), none of the investigated Ti-based alloys formed a fully amorphous structure after casting or laser remelting. This can be attributed to both compositional factors and thermal conditions during processing. Although the studied alloys meet key GFA criteria such as atomic size mismatch and negative heats of mixing [36], they also contain Si, which promote the early crystallization of silicides (e.g., Ti_5_Si_3_) even under high cooling rates. In Ti–Fe–Si and Ti–Nb–Zr–Si systems, full amorphization is typically limited to ribbons or powders produced by rapid solidification [21,33]. Also Ti–Zr–Si–Ta systems were researched mostly in ribbon form [31,32].

Fully amorphous Ti-based BMGs have been produced via SLM (e.g., Ti_47_Cu_38_Zr_7.5_Fe_2.5_Sn_2_Si_1_Ag_2_) but only in systems with inherently higher GFA containing Cu [29]. In alloys with lower GFA, such as Ti_42_Zr_35_Si_5_Co_12.5_Sn_2.5_Ta_3_, partial crystallization occurs even under optimized SLM conditions [30]. In the present study, standard laser remelting likely did not provide sufficient cooling rates to fully suppress crystallization, particularly in alloys intentionally formulated without elements such as Ni, Be, or Cu (for biocompatibility reasons) and instead containing Si and β-stabilizing elements such as Zr, Nb, and Ta. However, the absence of strong glass-forming elements inherently lowers GFA [37]. Recent studies further demonstrate that achieving amorphous structures in such systems often requires specialized scanning strategies [38]. The crystallinity observed here is therefore consistent with known challenges in processing Ti-based BMGs of moderate GFA, but even in crystalized form, these alloys, e.g., Ti-Nb-Zr-Si, are seen as interesting for medical applications [39].

### 3.2. Crack Density

**Figure 6:**
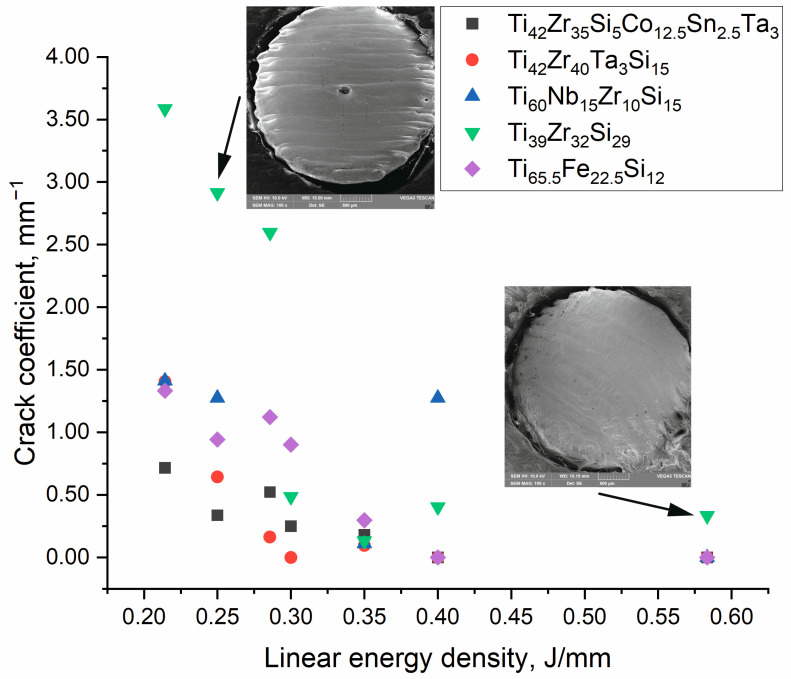
Crack coefficient in relation to linear energy density for as cast sample and SLM remelted samples for all alloys, inserts as follows: (i) top left with crack pattern on Ti_39_Zr_32_Si_29_ sample, and (ii) bottom right uncracked Ti_42_Zr_40_Ta_3_Si_15_ sample.

Figure 6 presents the measured crack density (crack length per area) of each alloy as a function of the laser energy density (with the as-cast condition included for reference). Cracks, when they occurred, appeared as long, straight fissures on the remelted surface, often following a pattern consistent with thermal contraction stresses. At the lowest energy input (0.21 J/mm, Table 1 row 2), all alloys showed some degree of cracking in the remelted tracks. The crack density was highest at *ED* = 0.21 J/mm (Table 1 row 2) for every alloy. This can be attributed to the extremely fast cooling and steep temperature gradients at low energies: a small, rapidly solidified melt pool can generate high tensile residual stresses as the material contracts, leading to brittle fracture in susceptible alloys. As the laser energy (and thus heat input) was increased, the crack density decreased in all cases. Higher *ED* means a larger and hotter melt pool, slower cooling rates, and more opportunity for stress relaxation or ductile flow, thereby mitigating cracking [40]. By around 0.30–0.35 J/mm (Table 1 rows 5–6), most alloys showed a decrease in cracks, and above ~0.40 J/mm (Table 1 row 7), cracks were completely eliminated in three of the five alloys. The T_i39_Zr_32_Si_29_ alloy was the most crack-prone alloy; it continued to exhibit some cracking even at the highest energy (0.58 J/mm, Table 1 row 8), although crack density was reduced at higher *ED*. This alloy’s inherently high brittleness (being essentially a silicide network) likely made it unable to accommodate thermal strains without cracking unless the heating was extremely slow. In contrast, the Ti_42_Zr_35_Si_5_Co_12.5_Sn_2.5_Ta_3_, Ti_42_Zr_40_Ta_3_Si_15_, and Ti_65.5_Fe_22.5_Si_12_ alloys were the most crack-resistant. Above ~0.4 J/mm there were no cracks on their remelted surface. The superior crack resistance of the Ti_42_Zr_35_Si_5_Co_12.5_Sn_2.5_Ta_3_, Ti_42_Zr_40_Ta_3_Si_15_, and Ti_65.5_Fe_22.5_Si_12_ alloys correlates with their lower hardness and reduced volume fraction of brittle phases, which are typically associated with higher ductility. The alloys Ti_42_Zr_40_Ta_3_Si_15_ and Ti_65.5_Fe_22.5_Si_12_ crystallize predominantly in the β-phase of titanium, which is known for its ductile and stress-accommodating nature [41]. The Ti_42_Zr_35_Si_5_Co_12.5_Sn_2.5_Ta_3_ alloy also contains a significant fraction of β-phase titanium, alongside σ-phase, and is rich in β-stabilizing elements such as Ta and Co. In contrast, although the Ti_60_Nb_15_Zr_10_Si_15_ alloy also shows a β + σ microstructure, it exhibits significantly higher hardness and elastic modulus, which reduces its capacity to relax thermal stress. On the other hand, the Ti_39_Zr_32_Si_29_ alloy exhibits a microstructure dominated by silicide phases, which are intrinsically hard and stiff [42].

As the present study involved remelting experiments on solid cast samples only cracking was analyzed. The melt pools fully covered the substrate; therefore, other defects typically associated with additive manufacturing such as lack of fusion, unmelted particles, and porosity [43] were not further investigated. Based on the results of this initial screening, additional experiments are planned using powder-bed systems, in which quantitative porosity analysis will be performed.

### 3.3. Hardness and E-Moduli

**Figure 7:**
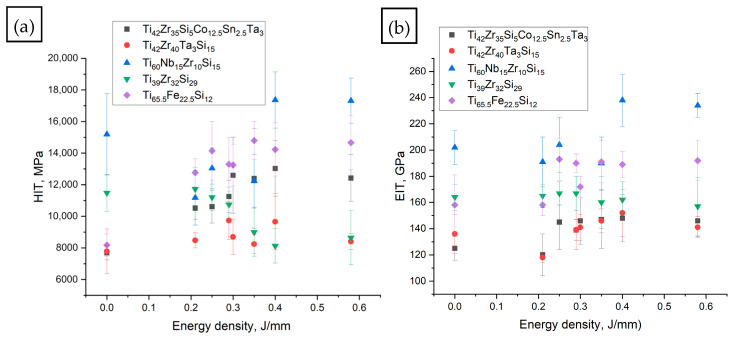
(**a**) Hardness and (**b**) elastic modulus in relation to linear energy density for as-cast samples and SLM remelted samples for all alloys.

The hardness values and Young’s moduli of the alloys are presented in Figure 7. The lowest Young’s modulus was obtained for Ti_42_Zr_35_Si_5_Co_12.5_Sn_2.5_Ta_3_, whereas the highest was measured for Ti_60_Nb_15_Zr_10_Si_15._ After laser remelting, the Young’s modulus slightly decreased at the lowest linear energy density (0.21 J/mm, Table 1 row 2) for Ti_42_Zr_35_Si_5_Co_12.5_Sn_2.5_Ta_3_, Ti_42_Zr_40_Ta_3_Si_15_, and Ti_60_Nb_15_Zr_10_Si_15_, and remained relatively stable for the remaining alloys. At higher energy densities, the modulus generally increased relative to the as-cast state and then stabilized. A different trend was observed for Ti_39_Zr_32_Si_29_, which showed an approximately constant modulus regardless of remelting energy or even a slight decrease at higher energy densities.

A similar trend was observed for hardness. The lowest hardness was recorded for Ti_42_Zr_40_Ta_3_Si_15,_ while the highest was found for Ti_60_Nb_15_Zr_10_Si_15_. At lower energy densities (0.21–0.35 J/mm, Table 1 rows 2–4), hardness increased initially and then stabilized at a higher level, except for Ti_39_Zr_32_Si_29_, where hardness decreased after remelting above 0.3 J/mm (Table 1 rows 6–8). It should be noted that both Young’s modulus and hardness exhibit considerable scatter due to microstructural and phase inhomogeneity.

In the literature, Ti_60_Nb_15_Zr_10_Si_15._ exhibits a hardness of 6.1–8.9 GPa and modulus of 94–144 GPa [17,33,44]. In this study, significantly higher values were obtained (~15 GPa and ~202 GPa) due to extensive silicide precipitation. Laser remelting slightly reduced the hardness to ~12–13 GPa, while the modulus remained high. For Ti_42_Zr_40_Ta_3_Si_15_, previous reports indicated a hardness of ~5.1 GPa and modulus of ~93 GPa [45], both of which are lower than those observed here, consistent with the presence of an amorphous structure in earlier studies. The observed increase in hardness and modulus after remelting is typical of annealed or nanocrystallized metallic glasses [46]. Comparable behavior was noted in Ti–Fe–Si alloys produced by pulsed current sintering, which exhibited α-Ti + Ti_5_Si_3_ microstructures and hardness around 707 HV (~7.6 GPa) [46], closely matching the present value (~8.2 GPa).

In summary, the fully crystalline microstructures with significant silicide precipitation resulted in higher elastic moduli and hardness values compared to typical amorphous Ti-based BMGs. The obtained values are higher than for standard Ti-6Al-4V (111–117 GPa [6]) or comparable for Ti_42_Zr_35_Si_5_Co_12.5_Sn_2.5_Ta_3_ (~120 GPa) and Ti_42_Zr_40_Ta_3_Si_15_ (~118 GPa) (after remelting 0.21 J/mm, Table 1 row 2).

### 3.4. Cytotoxicity Assessment

#### 3.4.1. Indirect Contact Method (Extract Test)

**Figure 8:**
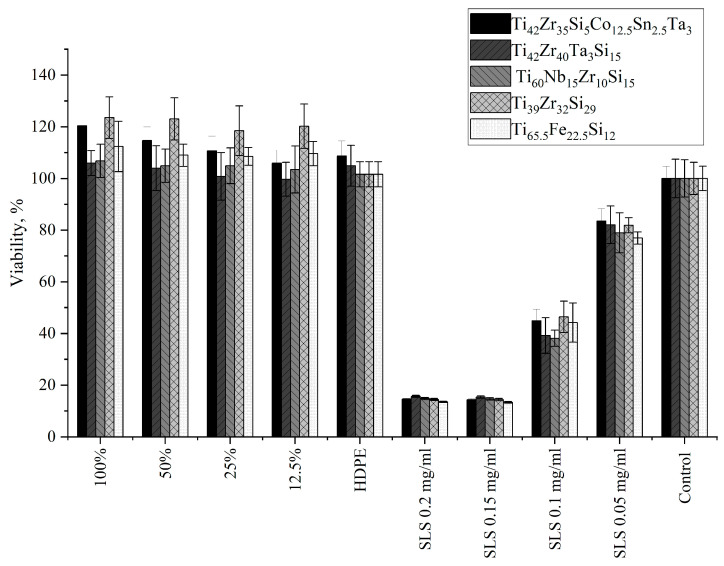
Viability of L929 fibroblasts after 24 h of exposure to the extract from alloys 1–5, which were previously polymerized for 24 h at 37 °C before extraction. Extract concentrations: 100%, 50%, 25%, 12.5%; negative control: HDPE; positive control: SLS at concentrations of 0.2, 0.15, 0.1, and 0.05 mg/mL; blank sample: reference control. Cell viability in both positive and negative controls confirms proper responsiveness of the cell culture. MTT assay.

**Figure 9:**
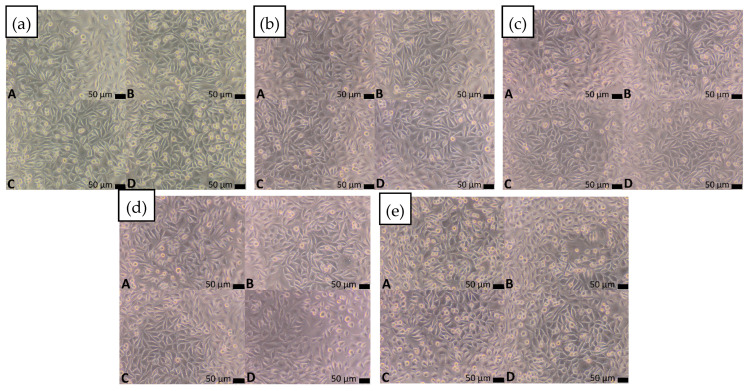
Indirect contact of L929 fibroblasts after 24 h of culture with extracts from materials (**a**) Ti_42_Zr_35_Si_5_Co_12.5_Sn_2.5_Ta_3_, (**b**) Ti_42_Zr_40_Ta_3_Si_15_, (**c**) Ti_60_Nb_15_Zr_10_Si_15_, (**d**) Ti_39_Zr_32_Si_29_, (**e**) Ti_65.5_Fe_22.5_Si_12_; (A–D): A: 100% extract, B: 50%, C: 25%, D: 12.5%, Magnification 100×.

Although no amorphous phase was achieved, cytotoxicity tests were still justified, as the alloys were specifically designed with biocompatible elements and could retain favorable biological performance in their crystalline state. Previous studies on crystallized Ti–Nb–Zr–Si systems have shown that they remain relevant for biomedical applications [39]. All tested alloys exhibited non-cytotoxic behavior, with cytotoxicity grade 0 based on PN-EN ISO 10993-5:2009 [34] (Table 2). The reactivity of the test system was verified using SLS (positive control), which induced a dose-dependent cytotoxic effect, and HDPE served as negative control (high-density polyethylene, United States Pharmacopeia (USP) Reference Standard, Sigma-Aldrich, St. Louis, MO, USA), which showed no adverse effect. These results confirm the sensitivity and reliability of the assay. Cell viability after 24 h exposure to material extracts is presented in Figure 8. Morphological analysis of L929 fibroblasts revealed no abnormalities for any of the tested materials. Importantly, none of the materials reduced cell viability compared to the blank control Figure 9. Moreover, extracts from materials Ti_42_Zr_35_Si_5_Co_12.5_Sn_2.5_Ta_3_, Ti_39_Zr_32_Si_29_, and Ti_65.5_Fe_22.5_Si_12_ significantly increased cell proliferation, as confirmed by statistical analyses (Table 2). Statistical analyses included the Shapiro–Wilk test (*p* > 0.05 for all groups), followed by one-way ANOVA and Tukey’s post hoc test (significance level *p* < 0.05), which is presented in Table 2. Only statistically significant differences relevant to material evaluation were reported.

#### 3.4.2. Direct Contact Method

**Figure 10:**
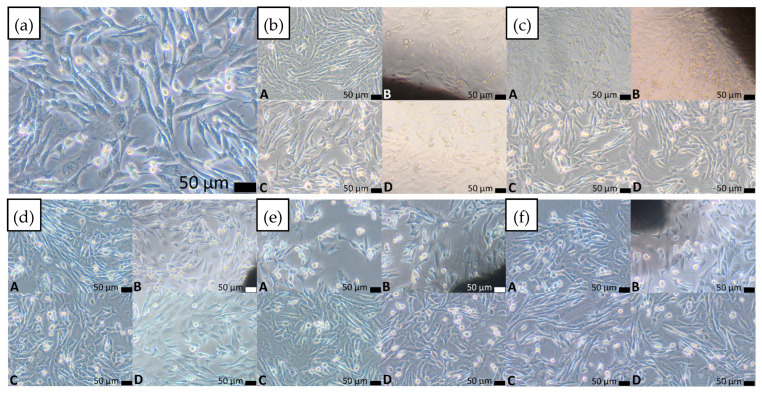
(**a**) Control culture without contact with the tested materials shows normal Balb/3T3 cell morphology. Direct contact of Balb/3T3 fibroblasts after 24 h with materials: (**b**) Ti_42_Zr_35_Si_5_Co_12.5_Sn_2.5_Ta_3_, (**c**) Ti_42_Zr_40_Ta_3_Si_15_, (**d**) Ti_60_Nb_15_Zr_10_Si_15_, (**e**) Ti_39_Zr_32_Si_29_, (**f**) Ti_65.5_Fe_22.5_Si_12_; (A–D): (A) under the sample, (B) at the sample edge, (C) at a distance of 1 cm from the sample, (D) farther away from the sample. Magnification 100×.

The direct contact assay evaluated cell morphology (Balb/3T3 fibroblasts) based on a 0–4 reactivity scale. All materials scored 0, indicating no cytotoxic effect. Fibroblasts under and around the samples maintained normal morphology, comparable to the control (Figure 10). No morphological changes indicative of cytotoxicity were observed.

According to PN-EN ISO 10993-5:2009 [34], a cytotoxic effect is considered to be present if cell viability is reduced by at least 30% or if morphological changes reach grade 2 or higher. The conducted studies—both using the indirect contact method (MTT assay) and the direct contact method (cell morphology assessment)—did not meet these criteria for any of the tested materials. All alloys were classified as non-cytotoxic (cytotoxicity grade 0), indicating no adverse effects on cells under in vitro conditions.

Statistical analysis of MTT assay (indirect contact) revealed that all tested alloys were non-cytotoxic. Moreover, extracts from materials Ti_42_Zr_35_Si_5_Co_12.5_Sn_2.5_Ta_3_, Ti_39_Zr_32_Si_29_, and Ti_65.5_Fe_22.5_Si_12_ even enhanced fibroblast proliferation compared with the control (*p* < 0.01), indicating excellent biocompatibility (Table 2).

It is also noteworthy that extracts from the alloys Ti_42_Zr_35_Si_5_Co_12.5_Sn_2.5_Ta_3_ and Ti_39_Zr_32_Si_29_ exhibited increased cell proliferation, which may be related to the specific chemical composition of these alloys. These findings are consistent with the results of the direct contact method, which likewise showed no morphological changes indicative of cytotoxicity. Given the positive biological response observed, these alloys remain promising for biomedical coatings or components where amorphous structure is not a prerequisite.

All investigated systems contained elements known for their high biological tolerance, such as titanium (Ti), zirconium (Zr), niobium (Nb), and tantalum (Ta). The literature highlights their favorable biocompatibility profiles and lack of cytotoxic effects. No additives with potential cytotoxic activity were used in this study, which may explain the positive outcomes of the tests conducted in accordance with PN-EN ISO 10993-5:2009 [47].

## 4. Conclusions

Five Ti-based alloys free of Ni, Be, and Cu solidified into crystalline α/β-Ti matrices containing Ti/Zr-based silicides, confirming their limited glass-forming ability at bulk scale. The initial goal of promoting amorphization via laser remelting was not met under the single-track SLM conditions used; standard processing did not reach the cooling rates necessary to produce a fully amorphous phase. Nevertheless, this work serves as an important screening step prior to powder-bed AM.

Laser remelting effectively refined the microstructures. A threshold energy density of ~0.4 J/mm was identified, above which most alloys solidified crack-free, particularly Ti_42_Zr_35_Si_5_Co_12.5_Sn_2.5_Ta_3_, Ti_42_Zr_40_Ta_3_Si_15_, and Ti_65.5_Fe_22.5_Si_12_. The alloys exhibited high hardness (7.6–15.2 GPa) and moduli of 125–202 GPa in a cast state, with mostly moderate increases after remelting due to microstructural refinement. Among them, in a cast state, Ti_42_Zr_35_Si_5_Co_12.5_Sn_2.5_Ta_3_ achieved the lowest modulus (~125 GPa), approaching that of Ti-6Al-4V, while Ti_60_Nb_15_Zr_10_Si_15_ was the stiffest (~200 GPa).

All compositions demonstrated excellent cytocompatibility, maintaining or enhancing fibroblast viability and confirming the absence of toxic ion release. Combining structural integrity, processability, and biocompatibility, Ti_42_Zr_40_Ta_3_Si_15_ emerged as the most promising candidate for further testing, although it must be emphasized that the amorphous structure was not achieved with tested laser parameters.

In summary, this study demonstrates the challenge of achieving amorphization in biocompatible, toxic-element-free Ti-based alloys using standard additive manufacturing, as the required cooling rates cannot be reached during remelting of bulk components. Further work should therefore focus on advanced scanning strategies and thermal management to enhance glass formation. Despite their crystalline structure, the investigated alloys may still be suitable for biomedical coatings due to their favorable biocompatibility, e.g., in form of coatings, although comprehensive evaluation of corrosion resistance and long-term stability is still required.

## Figures and Tables

**Table 1:** Summary of surface remelting parameters and corresponding linear energy density (ED) values.

Lp	Laser Power, W	Scanning Speed, mm/s	ED, J/mm
1	as cast	-	0
2	300	1400	0.21
3	350	1400	0.25
4	400	1400	0.29
5	300	1000	0.30
6	350	1000	0.35
7	400	1000	0.40
8	350	600	0.58

**Table 2:** Cytotoxicity grades for the extract test (according to PN-EN ISO 10993-5:2009 [34]) and comparison of average cell viability (V%) after exposure to 100% extracts from materials relative to the control (100% of cells in medium). Statistical significance assessed using Tukey’s test.

Material	Cytotoxicity Score	Description of Changes in Cell Cultures	V% (Mean)—Extract (100%)	V% (Mean)—Control	*p*-Value
Ti_42_Zr_35_Si_5_Co_12.5_Sn_2.5_Ta_3_	0	Single intracytoplasmic granules; no cell lysis observed; no inhibition of cell growth	120.36	100.00	0.000161
Ti_42_Zr_40_Ta_3_Si_15_	0	Single intracytoplasmic granules; no cell lysis observed; no inhibition of cell growth	105.95	100.00	0.579065
Ti_60_Nb_15_Zr_10_Si_15_	0	Single intracytoplasmic granules; no cell lysis observed; no inhibition of cell growth	106.79	100.00	0.255255
Ti_39_Zr_32_Si_29_	0	Single intracytoplasmic granules; no cell lysis observed; no inhibition of cell growth	123.53	100.00	0.000160
Ti_65.5_Fe_22.5_Si_12_	0	Single intracytoplasmic granules; no cell lysis observed; no inhibition of cell growth	112.38	100.00	0.003157

## Data Availability

The original contributions presented in the study are included in the article, further inquiries can be directed to the corresponding author: aleksandra.malachowska@pwr.edu.pl.
